# Impact of Self-Directed Multimodal Digital Training on Veterinary Students’ Ability to Interpret Equine Heart Sounds

**DOI:** 10.3390/vetsci13090981

**Published:** 2026-09-17

**Authors:** Chiara Bozzola, Dania Cingottini, Francesca Bindi, Micaela Sgorbini, Tommaso Vezzosi, Enrica Zucca

**Affiliations:** 1 Department of Veterinary Medicine and Animal Science, Università Degli Studi di Milano, 26900 Lodi, Italy; chiara.bozzola@unimi.it; 2 Department of Veterinary Sciences, Università di Pisa, San Piero a Grado, 56122 Pisa, Italy; dania.cingottini@phd.unipi.it (D.C.); francesca.bindi@phd.unipi.it (F.B.); micaela.sgorbini@unipi.it (M.S.); tommaso.vezzosi@unipi.it (T.V.)

**Keywords:** veterinary education, clinical skills training, self-directed learning, cardiac auscultation, digital phonocardiography

## Abstract

Cardiac auscultation is a fundamental clinical skill for veterinary graduates; however, acquiring this competence remains challenging because opportunities for repeated exposure to a wide range of cardiac abnormalities may often be limited during undergraduate training. This study evaluated whether supplementing written self-directed learning with digital equine phonocardiographic recordings improves veterinary students’ ability to recognize and interpret equine cardiac auscultation findings. Forty-one fourth-year veterinary students from the Universities of Milan and Pisa were voluntarily enrolled and randomly assigned to receive either written learning materials alone (Group 1, *n* = 21) or the same materials supplemented with digital phonocardiographic recordings (Group 2, *n* = 20). Participants completed a baseline test (T0). After a four-week self-directed learning period, 30 students (Group 1 = 15; Group 2 = 15) completed the post-training test (T1) and were included in the final analysis. The statistical analysis provided no significant differences for the total score (*p* = 0.064) or Q1 score (*p* = 0.349), whereas a significant difference was observed for Q2 score (*p* = 0.039), showing greater improvement in Group 2. These findings suggest a potential educational benefit of supplementing written learning materials with digital phonocardiographic recordings, particularly for the ability to distinguish among different cardiac abnormalities.

## Simple Summary

Cardiac auscultation is an essential clinical skill for veterinary students, but learning to recognize normal and abnormal heart sounds can be challenging. Since exposure to a wide variety of cardiac abnormalities may be limited during undergraduate training, digital learning resources may allow students to repeatedly practice with authentic clinical recordings outside the clinical setting. In this study, students provided with written learning materials supplemented with digital equine phonocardiographic recordings showed greater improvement in the ability to distinguish among different cardiac abnormalities than those using written materials alone. These findings suggest that digital phonocardiographic recordings may represent useful complementary educational resources alongside traditional clinical teaching for supporting the acquisition of cardiac auscultation skills.

## 1. Introduction

Cardiac auscultation is a fundamental component of the veterinary physical examination and is recognized as a Day One competency for graduating veterinarians [1,2]. It provides an inexpensive, rapid, and non-invasive method for identifying normal and abnormal heart sounds, including murmurs and arrhythmias, thereby guiding decisions regarding further diagnostic investigations, such as electrocardiography and/or echocardiography. Despite advances in diagnostic imaging, accurate auscultation remains an essential clinical skill in both small and large animal practice and continues to play a key role in the initial assessment of cardiovascular disease [3].

Acquiring competence in cardiac auscultation, however, is challenging. It requires the integration of auditory perception, pattern recognition, and interpretation of physiological and pathological findings rather than simply acquiring theoretical knowledge. These competencies are developed through repeated practice and exposure to a wide variety of cardiac sounds [4,5]. More broadly, recent equine-specific evidence indicates that the reliability of interpreting clinical indicators may vary according to the type and complexity of the information assessed and the level of observer preparation [6]. Several studies have shown that veterinary students struggle to recognize and correctly interpret heart sounds, and that these difficulties may persist even after graduation. Naylor and colleagues reported that veterinary students correctly identified only 29% of equine cardiac recordings, while general practitioners achieved only modestly higher diagnostic accuracy, highlighting the need for improved educational strategies [7]. More recently, Mullowney and colleagues found that recent veterinary graduates did not perform better than final-year veterinary students in recognizing canine cardiac auscultation findings, suggesting that auscultation skills do not necessarily improve during the first years of clinical practice [2].

Developing proficiency in cardiac auscultation during veterinary training is inherently difficult because opportunities for deliberate practice may be limited. In fact, exposure to appropriate clinical cases depends on patient availability, case diversity, and scheduling constraints, reducing students’ opportunities to repeatedly encounter pathological cardiac findings [8]. These challenges are particularly relevant in equine medicine, where access to horses presenting a wide spectrum of murmurs and arrhythmias may be limited during undergraduate clinical rotations [8]. Consequently, there has been increasing interest in educational strategies that extend learning beyond traditional face-to-face teaching and promote self-directed practice within clinical skills laboratories, an approach that has been explored using objective assessments of clinical performance in veterinary students [9].

Recent advances in educational technology have expanded opportunities to teach auscultation using digital resources [4,5]. Digital stethoscopes allow high-quality recordings of heart sounds, while digital phonocardiograms provide a visual representation of acoustic signals and, when synchronized with electrocardiographic tracings, facilitate the correlation between audible cardiac events and electrical activity [10,11]. Educational research has shown that repeated exposure to cardiac sounds can improve students’ ability to recognize cardiac murmurs [4]. In addition, integrating auditory and visual information through digital phonocardiography may facilitate the interpretation of cardiac sounds by allowing learners to associate acoustic findings with their corresponding graphical representations [12]. In veterinary medicine, Balogh and colleagues demonstrated that additional self-directed or remote training using digital phonocardiograms improved veterinary students’ ability to recognize canine heart murmurs and refine the diagnosis of arrhythmias [5]. Similarly, Wood and colleagues recently developed an audio-visual learning resource for equine cardiac auscultation and reported improvements in students’ confidence and perceived competence after using the resource [8].

Despite these encouraging findings, the specific contribution of digital phonocardiographic recordings to objectively measured learning outcomes in equine cardiac auscultation remains unclear. Previous studies have explored digital phonocardiography or audio-visual resources in veterinary education. However, adding digital recordings to conventional written self-directed learning provides additional benefits for measured student auscultation skills has not been clearly established. Therefore, the aim of the present study was to compare the effectiveness of conventional written learning materials alone or supplemented with digital equine phonocardiographic recordings in improving veterinary students’ ability to interpret normal and abnormal equine cardiac auscultation findings.

## 2. Materials and Methods

### 2.1. Human Ethical Approval

The study protocol involving veterinary medicine students was reviewed and approved by the Institutional Ethics Committee of the University of Milan (No. 122/23) and the Bioethics Committee of the University of Pisa (No. 1/2024). All participants provided informed consent prior to enrolment.

### 2.2. Digital Tracing Recording

Digital phonocardiographic recordings (including the electrocardiogram (ECG), heart sound, and phonocardiogram) have been previously collected from horses undergoing clinical examinations for diagnostic purposes. All recordings were acquired using a single-lead smartphone-based digital stethoscope (Eko DUO Digital Stethoscope, Eko Devices Inc., Oakland, CA, USA) and stored online on a dedicated website (Eko Dashboard) [13].

To be suitable for educational purposes, the recordings were selected based on the clarity of the relevant auscultation finding and the absence of substantial background noise, including excessive interference caused by external sounds or movement of the stethoscope against the animal’s skin, by two experienced veterinarians with expertise in equine cardiology, one of whom was an ECEIM Diplomate.

The audio of the selected recordings was digitally amplified by a technician solely to increase their volume and improve audibility through headphones during the tests. No noise reduction or other audio processing was applied. All participants were provided with the same model of wired headphones connected to the computers via a standard audio jack.

### 2.3. Student Population

Fourth-year veterinary students from the Universities of Veterinary Medicine of Milan and Pisa were voluntarily enrolled in the present study. The inclusion criterion was completion of both the baseline (T0) and post-training (T1) tests.

As part of the fourth-year veterinary curriculum, all students had previously completed the Equine Clinical Propaedeutics course, which included theoretical lectures and practical training on the physical examination of the equine cardiovascular system. Each student was assigned unique login credentials (ID and password), which ensured anonymity throughout the study while allowing individual performance to be tracked.

### 2.4. Study Design

All participants completed a baseline test (T0) followed by a second test (T1) after a four-week period of self-directed learning. The tests were supervised by an investigator who had access only to anonymous participant IDs and was blinded to the participants’ personal identities, group allocation, and the learning intervention received by each participant. Participants were informed that the test was conducted for research purposes only and had no impact on their academic evaluation; consequently, any dishonest behavior would provide no personal benefit while potentially compromising the validity of the study findings.

At T0, all participants completed an online test designed to evaluate their baseline ability to recognize and interpret equine cardiac auscultation findings. The test was completed individually on computers in the University computer laboratory under standardized testing conditions. The test was developed using the Moodle learning management system and administered through Safe Exam Browser (SEB). Participants had a two-hour period to complete the test.

Each student accessed the test using previously assigned login credentials ensuring their anonymity throughout the whole study. The investigators responsible for data analysis perfomed at the end of the study had access only to anonymous participant IDs and their group allocation.

The baseline test consisted of 18 multiple-choice questions based on 10 audio recordings, including 2 normal heart sounds, 4 arrhythmias, and 4 heart murmurs. After submitting their answer to the question, participants could not return to modify their response.

The 18 questions included one initial classification question (Q1 question) for each of the 10 recordings and one additional classification question (Q2 question) for each recording containing an arrhythmia (*n* = 4) or a heart murmur (*n* = 4). The test was based exclusively on audio recordings (mp3 files), without access to the corresponding ECG or phonocardiogram. Recordings containing both an arrhythmia and a heart murmur simultaneously were not included. Participants were free to replay each recording an unlimited number of times before answering the question.

For each recording, participants were first asked to classify the finding as a normal heart sound, arrhythmia, or heart murmur (Q1 question). Depending on the underlying diagnosis of the recording, participants were then presented with a second question (Q2 question) requiring further classification of the abnormality (arrhythmia or murmur). Regardless of their response to Q1, participants were subsequently presented with the corresponding Q2 question, which specified whether the audio recording contained an arrhythmia or a heart murmur and required them to further classify the type of cardiac abnormality. For recordings containing an arrhythmia, students were asked to identify the type of arrhythmia (atrial fibrillation, sinoatrial/atrioventricular block, or premature complex). For recordings containing a heart murmur, students were asked to classify the murmur as systolic, diastolic, or continuous (Table 1). The Q2 questions explicitly indicated whether the previous recording contained an arrhythmia or a heart murmur and invited participants to listen to the recording again before classifying the abnormality. Recordings representing normal heart sounds were not followed by a Q2 question. An “*I don’t know*” response option was available for all questions to minimize guessing.

Following enrollment in the study, a technician assigned each participant a sequential number according to the order of enrollment, separately for each university. These numbers were entered into a web-based randomization tool [14], which randomly allocated participants from each university as evenly as possible between the two study groups. Following randomization, each participant was assigned an anonymous study ID and, after completing the T0 test, received the learning materials corresponding to their group allocation.

Participants were informed about the different learning resources available to the two study groups and were therefore aware of the learning approach to which they had been assigned; consequently, participants were not blinded to group allocation. Both groups received the same written theoretical material covering the principles of equine cardiac auscultation, including physiological heart sounds, cardiac arrhythmias, and heart murmurs [15,16].

Group 1 followed a self-directed learning pathway based exclusively on the provided theoretical material and did not have access to additional practical resources.

Group 2 followed the same self-directed learning pathway as Group 1 and, in addition, had access to digital phonocardiographic recordings obtained from real clinical cases and available at the University Skills Laboratory (Skills Lab). Each recording displayed the ECG and phonocardiogram, allowing simultaneous playback of the corresponding heart sounds. The Skills Lab was accessible from Monday to Friday afternoons, allowing students to independently schedule practice sessions during the four-week study period. The frequency and duration of practice sessions in the Skills Lab were not recorded. The available material consisted of 6 digital phonocardiographic recordings acquired using the Eko DUO digital stethoscope, including 2 normal sinus rhythms, 2 arrhythmias, and 2 heart murmurs. These recordings were representative of the types of cardiac findings included in the tests but were different from the recordings used in the assessments. The self-learning materials were made available to students for self-directed learning throughout the four-week study period.

Following this training period, the participants completed a second test (T1) using their anonymous login credentials.

The T1 test was conducted under the same conditions as the T0 test. The T1 test consisted of the same 18 multiple-choice questions, presented in a different randomized order. To reduce the possibility of a recognition or test–retest effect, students were not informed that the same audio recordings would be used in the T1 assessment and were therefore unaware at T0 that they would subsequently be re-exposed to the same recordings.

At the end of the T1 test, participants completed a short questionnaire to evaluate their experience with the training activity. The questionnaire included four questions assessing: the amount of time spent using the training material, categorized as less than 1 h, 1–2 h, 2–4 h, or more than 4 h; whether the activity was perceived as potentially beneficial for teaching; whether similar activities should be introduced into other subjects; and overall satisfaction with the training, rated on a 0–5 scale, with higher scores indicating greater satisfaction.

### 2.5. Test Scoring

Students received 1 point for each correct answer. For each test, three scores were calculated: a total score (out of 18 points), a Q1 question score (out of 10 points), and a Q2 question score (out of 8 points). The Q1 score reflected the ability to correctly identify the cardiac finding (normal heart sound, arrhythmia, or heart murmur), whereas the Q2 score reflected the ability to further classify arrhythmias and heart murmurs. All participants were allowed to answer the Q2 questions regardless of whether the corresponding Q1 question had been answered correctly. Students did not receive feedback on their tests’ score.

### 2.6. Statistical Analysis

An “a priori” power analysis was not performed, as participant recruitment was voluntary and the available sample size was determined by student enrollment during the study period.

The Shapiro–Wilk test was used to assess data distribution. Normally distributed data were reported as mean and standard deviation, whereas non-normally distributed data were reported as median, minimum, and maximum. Qualitative variables were reported as frequencies and percentages.

Baseline scores for the total test score, as well as for Q1 and Q2 questions, were analyzed using the Mann–Whitney U test to assess whether the two groups (Group 1 and Group 2) differed in their baseline knowledge before the training period, regardless of the university to which the students belonged.

To directly assess whether changes in performance over time differed between the two educational groups, a repeated-measures general linear model was performed separately for the total, Q1, and Q2 scores. Time (T0 and T1) was included as the within-subject factor and group (Group 1 and Group 2) as the between-subject factor. The Group × Time interaction was used to assess whether the magnitude of change from T0 to T1 differed between the two groups. Partial eta squared (η^2^p) was reported as the effect size for the interaction. In addition, individual change scores (T1–T0) were calculated for each participant, and the mean difference in change between Group 2 and Group 1 was estimated for each outcome. To quantify the precision of these between-group differences in change, 95% BCa bootstrap CIs based on 2000 bootstrap samples were calculated.

Given the observed difference in baseline total scores between the two groups, an additional ANCOVA was performed as a baseline-adjusted sensitivity analysis, with the T1 total score as the dependent variable, group as the fixed factor, and T0 total score as the covariate. Homogeneity of regression slopes and homogeneity of error variances were assessed before interpreting the model.

As complementary analyses, for each group, changes in performance between T0 and T1 were assessed using the Wilcoxon signed-rank test. Analyses were conducted on the total test score and separately on Q1 and Q2 question scores. Effect sizes were calculated as r = |Z|/√N, where Z represents the standardized test statistic and N the number of paired observations. To quantify the precision of the estimated within-group changes, 95% bias-corrected and accelerated (BCa) confidence intervals (CIs) for the mean change (T1–T0) were estimated using 2000 bootstrap samples.

For the questionnaire data, differences in training satisfaction between groups were assessed using the Mann–Whitney U test.

A *p*-value < 0.05 was considered statistically significant.

## 3. Results

### 3.1. Student Population

Forty-one fourth-year veterinary students from the Universities of Veterinary Medicine of Milan and Pisa were voluntarily enrolled in the present study. The students were randomized into Group 1 (21/41, 51.2%) and Group 2 (20/41, 48.8%). The analysis was conducted as a complete-case analysis, including only students who completed both T0 and T1 assessments.

Eleven students (11/41, 26.8%), 6 students (6/21, 28.6%) from Group 1 and 5 students (5/20, 25.0%) from Group 2, were excluded from the final analysis because they did not participate in the post-training assessment (T1).

Consequently, the final study population consisted of 30 students (30/41, 73.2%), with 15 students included in each group (Supplementary Figure S1). Among the students included in the finals analysis, Group 1 included 10 students (10/15, 66.7%) from the University of Milan and 5 students (5/15, 33.3%) from the University of Pisa; while Group 2 included 11 students (11/15, 73.3%) from the University of Milan and 4 students (4/15, 26.7%) from the University of Pisa.

### 3.2. Descriptive Statistics

**Figure 1:**
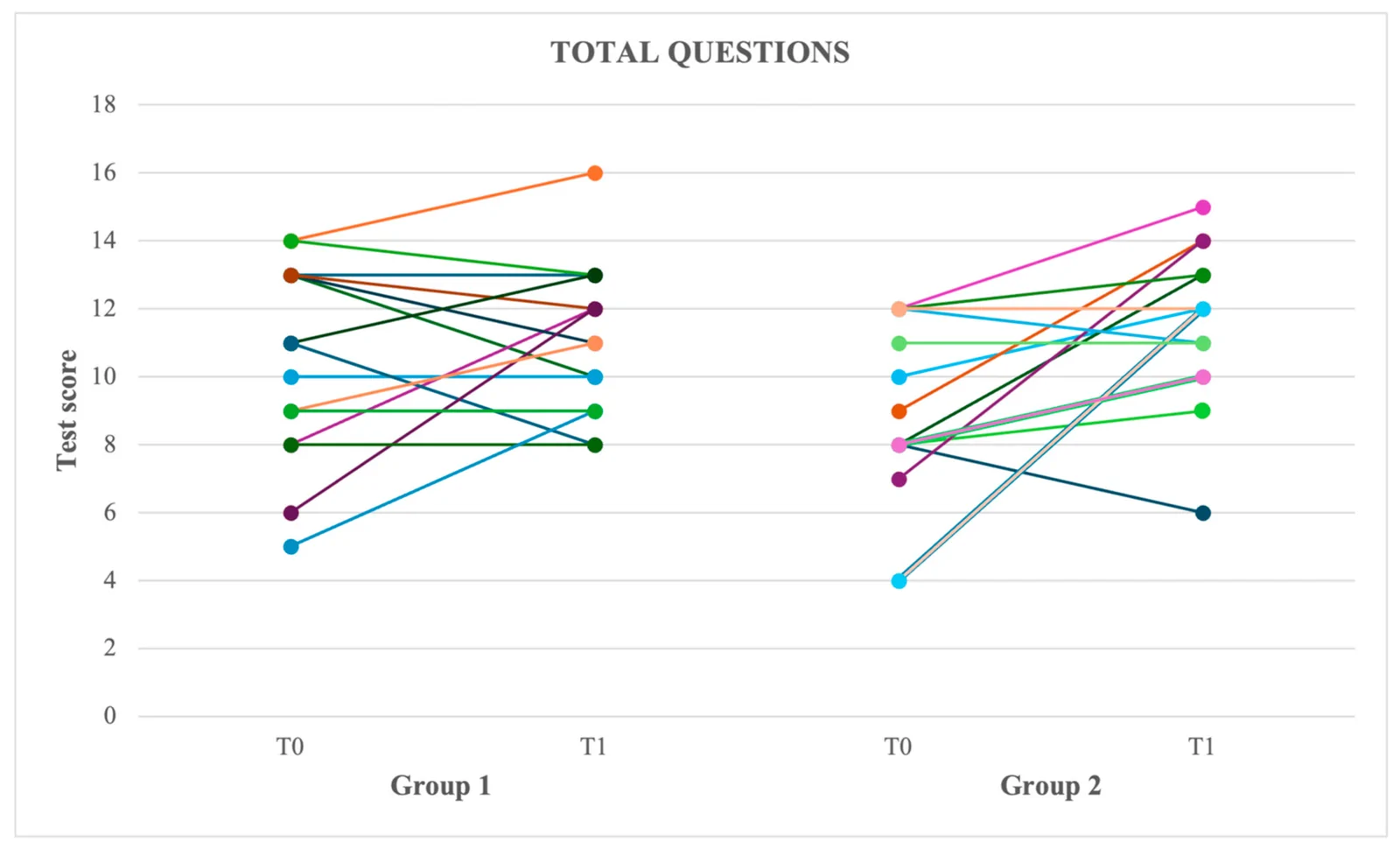
Individual changes in total question score (out of 18) between T0 and T1 in Group 1 and Group 2. Each line represents an individual participant and connects their scores at the two assessment time points. Overlapping trajectories may occur when participants obtained identical scores at both time points.

**Figure 2:**
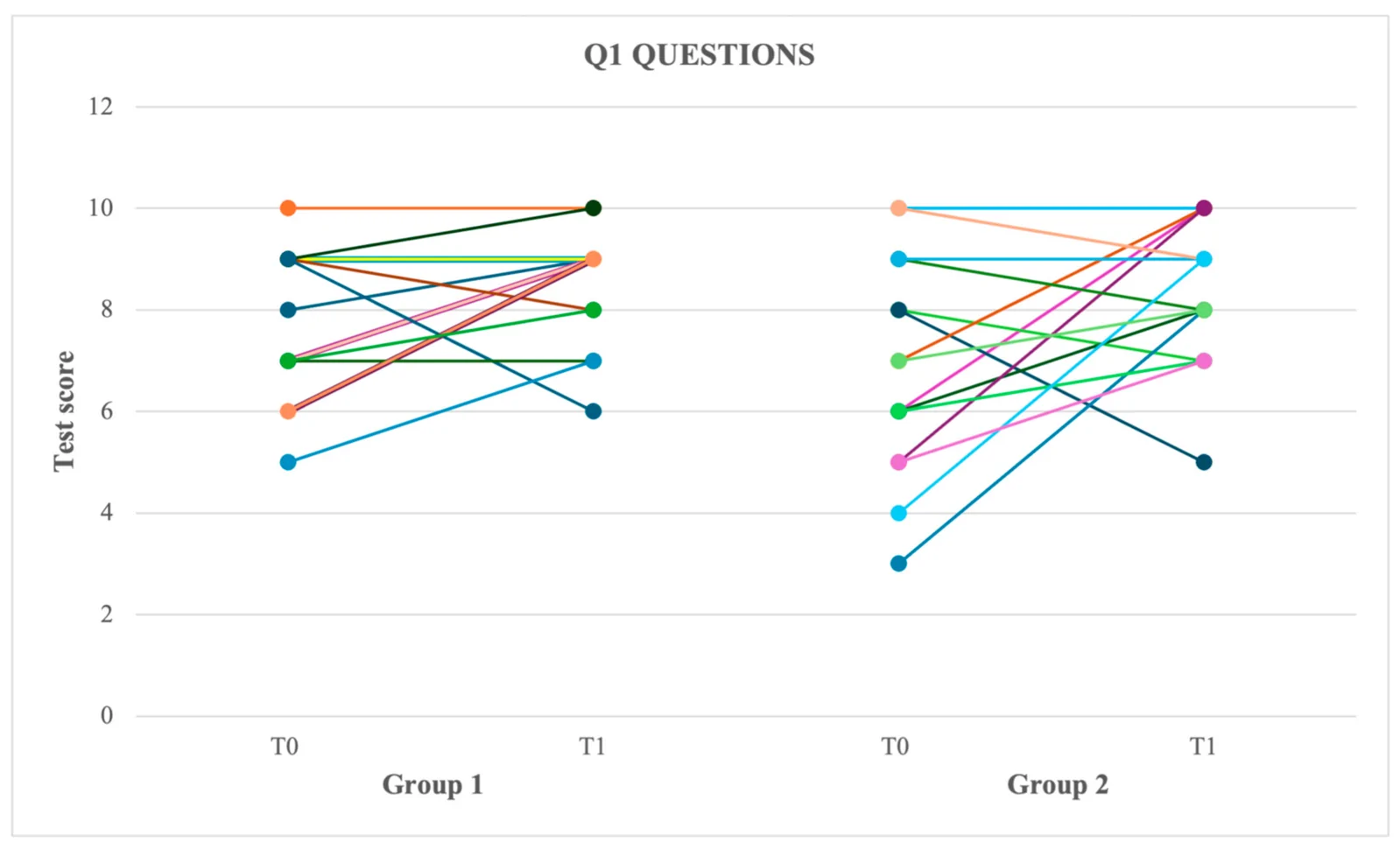
Individual changes in Q1 question score (out of 10) between T0 and T1 in Group 1 and Group 2. Each line represents an individual participant and connects their scores at the two assessment time points. Overlapping trajectories may occur when participants obtained identical scores at both time points.

**Figure 3:**
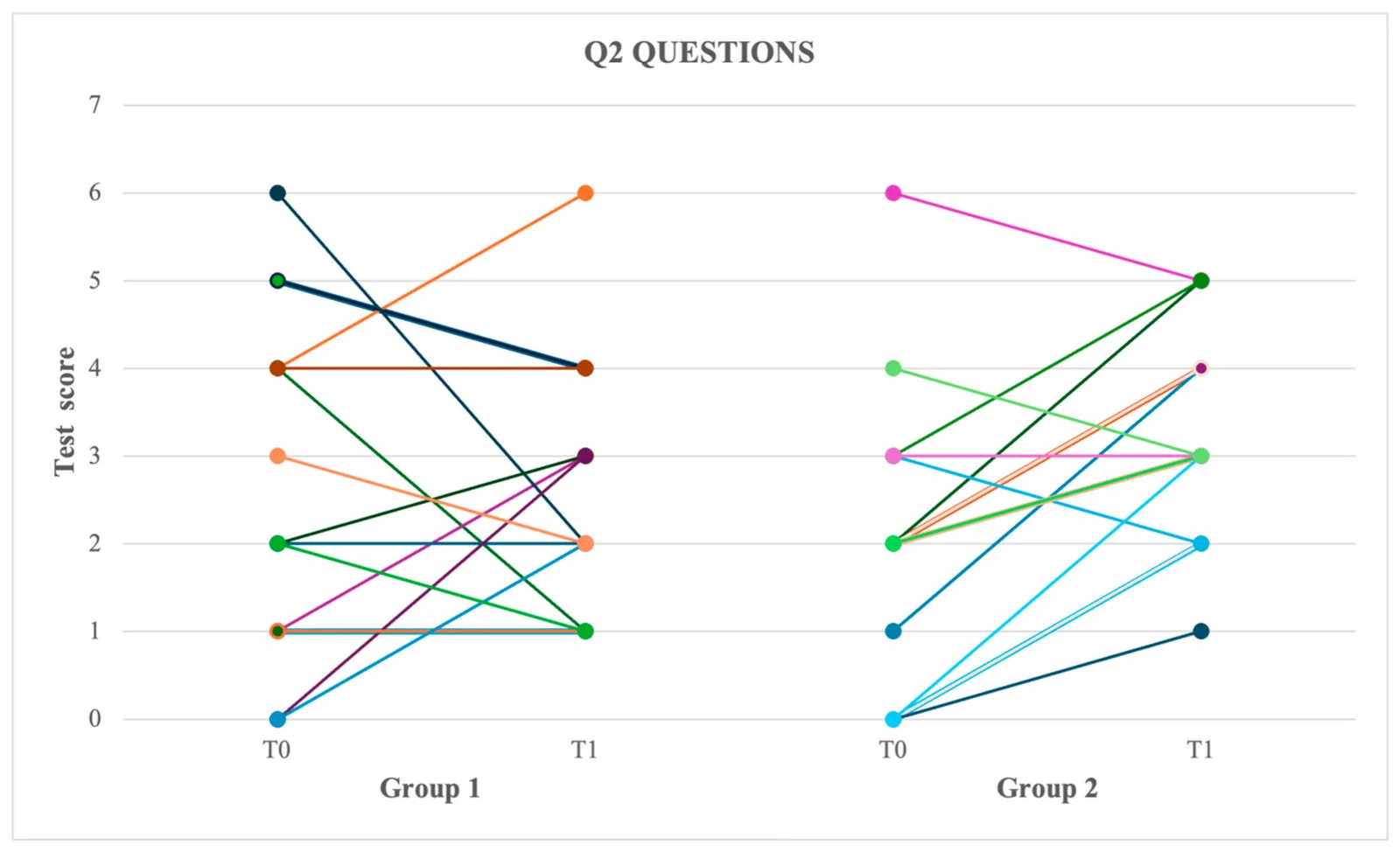
Individual changes in Q2 question score (out of 8) between T0 and T1 in Group 1 and Group 2. Each line represents an individual participant and connects their scores at the two assessment time points. Overlapping trajectories may occur when participants obtained identical scores at both time points.

Descriptive statistics for the total, Q1, and Q2 scores at T0 and T1 are presented in Table 2. Individual changes between T0 and T1 tests in total, Q1, and Q2 question scores are illustrated in Figure 1, Figure 2 and Figure 3, respectively, allowing visualization of the distribution and direction of individual changes in each group.

Participants’ responses to the qualitative questionnaire, completed at the end of the T1 assessment, are presented in Supplementary Table S1.

### 3.3. Statistical Results

Group 2 showed a lower median total score at T0 than Group 1 (8 vs. 11); however, the between-group baseline differences did not reach statistical significance (*p* = 0.098). Similarly, no statistically significant baseline differences were detected for Q1 (*p* = 0.233) or Q2 (*p* = 0.367) question score.

To account for the observed baseline difference in the total score, an additional baseline-adjusted analysis was performed. After adjustment for the T0 total score, no significant difference in T1 total score was observed between groups (*F*(1,27) = 1.21, *p* = 0.282, η^2^p = 0.043). The adjusted mean T1 scores were 10.92 (95% CI: 9.76–12.08) for Group 1 and 11.81 (95% CI: 10.66–12.97) for Group 2.

The Group × Time interaction was not statistically significant for the total score (*F*(1,28) = 3.73, *p* = 0.064, η^2^p = 0.118) or for the Q1 score (*F*(1,28) = 0.91, *p* = 0.349, η^2^p = 0.031). In contrast, a significant Group × Time interaction was observed for the Q2 score (*F*(1,28) = 4.67, *p* = 0.039, η^2^p = 0.143), with Group 2 showing a greater improvement over time than Group 1. Between-group differences in change and their 95% BCa confidence intervals are reported in Table 3.

Within Group 1, no significant differences were observed between T0 and T1 for the total test score (*p* = 0.347), for the Q1 question score (*p* = 0.099), or for the Q2 question score (*p* = 0.964). In contrast, Group 2 showed significant improvements between T0 and T1 in the total test score (*p* = 0.008), in the Q1 question score (*p* = 0.049), and in the Q2 question score (*p* = 0.007). These within-group analyses were considered complementary to the Group × Time analysis.

Training satisfaction was significantly higher in Group 2 than in Group 1 (*p* = 0.018), with median scores of 4 (3–5) and 3 (1–5) respectively.

## 4. Discussion

Cardiac auscultation differs from many other components of the physical examination because it is fundamentally a perceptual clinical skill rather than a purely cognitive one [2,4,17]. While theoretical knowledge is essential for understanding cardiac physiology, murmur timing, and arrhythmia mechanisms, accurate auscultation also requires learners to recognize acoustic patterns and associate them with specific pathological conditions. These abilities cannot be acquired through theoretical study alone but develop through repeated exposure to normal and abnormal heart sounds, allowing students to progressively refine their auditory discrimination skills [4,5].

Although Group 2 showed significant within-group improvements in the total, Q1, and Q2 scores, these findings should be interpreted in the context of the direct Group × Time analysis, which provided a more nuanced picture. A significant differential improvement between groups was observed only for Q2, suggesting a specific benefit of the digital intervention in the more detailed classification of cardiac murmurs and arrhythmias. These findings suggest that, although theoretical resources remain fundamental for understanding cardiovascular physiology and pathology [5], the addition of digital resources may provide complementary opportunities for developing practical auscultation skills, specially for the more detailed classification of arrhythmias and cardiac murmurs.

The observed pattern of improvement is consistent with current educational theories of clinical skill acquisition [4]. Digital phonocardiographic recordings provide learners with repeated access to authentic clinical examples, enabling them to revisit the same recordings as often as needed and focus on acoustic features that may be overlooked during a single clinical encounter [5,8]. This opportunity for self-paced practice may have contributed to the improvements observed in Group 2. However, because Group 2 differed from Group 1 in several aspects simultaneously, including access to authentic recordings, repeated listening, and simultaneous ECG and phonocardiographic visualization, the present study cannot determine the relative contribution of these individual components to the observed improvement. Therefore, the findings should be interpreted in relation to the combined digital learning intervention rather than for the isolated effect of digital phonocardiography or any single modality.

An additional advantage of digital phonocardiography is the simultaneous presentation of auditory and visual information. During the training period, students in Group 2 were able to observe the ECG tracing and phonocardiogram while listening to the corresponding heart sounds. Integrating these complementary sources of information may strengthen the association between audible cardiac events and their physiological origin [4,5,11]. More broadly, evidence from equine clinical assessment suggests that integrating complementary sources of clinical information may improve the consistency with which complex findings are interpreted, although the contribution of individual modalities remains context dependent [6]. Previous educational research has shown that combining auditory and visual modalities enhances learning more effectively than auditory information alone [4,17]. Although the present study was not designed to determine the relative contribution of repeated listening and visual support, our findings are consistent with the educational value of multimodal approaches for teaching cardiac auscultation.

Beyond improving individual learning, the findings of the present study may also have important implications for the organization of clinical teaching in veterinary curricula. Undergraduate students traditionally acquire auscultation skills through practical classes and clinical rotations involving real patients. Although this experience remains indispensable, it is inherently dependent on the clinical caseload available during the rotation [2,8]. As a result, students may not encounter a representative range of cardiac abnormalities, and exposure can vary considerably between cohorts. Some students may complete an equine medicine rotation without examining horses presenting with arrhythmias or clinically relevant murmurs, whereas others may encounter several such cases during the same training period. This unavoidable variability has been recognized as a limitation of workplace-based clinical education, emphasizing the need for complementary teaching strategies that provide more consistent learning opportunities [18].

Digital phonocardiographic recordings may offer a practical complementary approach to the variability inherent in clinical teaching by providing access to standardized learning materials that are independent of case availability. Unlike live patient encounters, digital recording libraries can preserve authentic clinical findings that remain accessible long after the patient has been discharged, potentially allowing students to experience the same clinically relevant cardiac abnormalities [8]. The potential use of digital recordings in veterinary education is supported by recent studies demonstrating the feasibility of smartphone-based digital stethoscopes for recording cardiac arrhythmias and murmurs in equine species [10,17]. In addition, these resources may promote self-directed learning, an educational approach increasingly encouraged within veterinary curricula [5,8]. Self-directed practice within clinical Skills Labs has also been explored in veterinary students [9], highlighting the potential of these settings to support independent clinical skills training. Digital recordings may therefore allow students to practice according to their individual learning needs rather than being limited to scheduled practical sessions. Importantly, digital recordings should be viewed as a complement rather than a replacement for hands-on clinical training and may support the development of core auscultation skills before their application in real patients. Developing these skills during undergraduate training may become increasingly relevant as digital auscultation technologies become increasingly available in veterinary practice, where recorded and amplified cardiac sounds may facilitate the identification and monitoring of cardiac abnormalities [19]. Because this approach can be implemented using relatively simple technology, a commercially available digital stethoscope and an online platform capable of storing synchronized heart sounds, phonocardiograms, and ECG tracings [10,11], educators may be able to develop their own libraries of digital cardiac recordings. These libraries could be incorporated into Skills Labs or shared through online learning platforms, potentially supporting both supervised practical training and independent study outside the classroom.

Our findings complement previous evidence from both veterinary and human medical education. In veterinary medicine, Balogh and colleagues demonstrated that remote access to digital phonocardiograms improved students’ ability to recognize canine heart murmurs and diagnose cardiac arrhythmias [5], while Wood and colleagues reported increased confidence and perceived competence following an audio-visual equine auscultation learning module [8]. Similarly, Bediang and colleagues showed that supplementing conventional theoretical teaching with digital heart sound recordings significantly improved knowledge acquisition in medical students [20]. While these studies assessed different educational outcomes, including objective diagnostic performance, knowledge acquisition, and self-reported confidence and competence, they collectively support the potential value of digital resources in undergraduate education.

Beyond their educational value, digital cardiac recordings may also have potential for future incorporation into Objective Structured Clinical Examinations (OSCEs) [21], offering a possible application for the assessment of students’ practical auscultation skills.

In addition to evaluating learning outcomes, students’ perceptions of the training were assessed using a structured questionnaire following the T1 assessment. All participants considered the activity potentially beneficial for teaching and indicated that similar activities should be implemented in other subjects. Moreover, students in Group 2 reported significantly higher satisfaction than those who received written learning materials alone. These findings suggest good acceptance of digital phonocardiographic recordings as a learning resource.

The present study has some limitations that should be acknowledged. First, recruitment was voluntary and the sample size was determined by student participation rather than an a priori power calculation; therefore, selection bias cannot be excluded. The present study should be considered a small exploratory study, and non-significant findings should not be interpreted as evidence of no effect or no difference. Although significant improvements were observed in the Group 2, larger multicenter studies involving students from multiple veterinary schools may increase the generalizability of these findings.

Given the limited and unbalanced number of participants from each institution, the potential influence of institution-specific differences in previous teaching exposure cannot be completely excluded. However, no statistically significant differences were detected between students from the two universities at T0.

Moreover, 11 of the 41 initially enrolled students (26.8%) did not complete the T1 assessment and were therefore excluded from the final complete-case analysis. Attrition was similarly distributed between the two groups (6 students from Group 1 and 5 from Group 2), resulting in an equal number of participants in the final groups (*n* = 15 each). Although this balanced attrition reduces concerns regarding differential loss between the two groups, the possibility of attrition bias cannot be completely excluded given the relatively small final sample size.

Another possible limitation is that the post-training assessment was performed only immediately after the four-week learning period. Therefore, the present study demonstrated short-term learning gains but did not assess long-term retention of auscultation skills.

The time dedicated to theoretical study and/or practical training in the Skills Lab was not recorded and was therefore not included in the statistical analysis to assess the relationship between the amount of training exposure and improvement in test performance.

The use of the same audio recordings and questions at T0 and T1 was chosen to allow direct comparison of performance on identical auscultation findings and to avoid potential differences in difficulty between tests. Students were not previously informed that the recordings used at T0 would be used at T1, the recordings available for Group 2 were representative of the types of cardiac findings included in the tests but were different from the recordings used in the assessments, and, finally, students did not receive feedback on their test answers after T0. However, a test–retest effect and a recognition of individual recordings cannot be completely excluded.

Finally, although the assessment instrument was specifically developed for the present study and did not undergo formal validation or pilot testing, the recordings were based on clinically confirmed diagnoses and were selected by experienced clinicians for the clarity of the relevant auscultatory findings and the absence of substantial background noise. Nevertheless, formal validation of the assessment instrument could further strengthen its use in future studies.

## 5. Conclusions

In conclusion, supplementing written self-directed learning with digital equine phonocardiographic recordings was associated with a pattern of greater short-term improvement in cardiac auscultation performance. However, the between-group difference in change did not reach statistical significance for the total score, or for Q1. In contrast, a significant Group × Time interaction was observed for Q2, suggesting a potential benefit of the combined digital learning material for the ability to distinguish among the different types of arrhythmias and cardiac murmurs.

Digital phonocardiographic recordings represent a practical educational resource that may complement traditional patient-based teaching. By providing repeated access to authentic clinical recordings independent of patient availability, these resources have the potential to reduce variability in students’ learning experiences and support the acquisition of one of the fundamental Day One clinical competencies expected of graduating veterinarians. Further studies with larger samples and standardized monitoring of students’ exposure to the digital recordings and assessment of long-term retention and performance during real-patient auscultation are warranted.

## Figures and Tables

**Table 1:** Pre-training test audio recordings and questions.

No. Audio Recordings	1	2	3	4	5	6	7	8	9	10
Q1	N	A	M	A	N	M	M	A	A	M
Q2	-	B	SM	AF	-	DM	SM	AF	B	SM

Abbreviations: No, number; Q1, first questions; Q2, second questions; A, arrhythmia; AF, atrial fibrillation; B, sinoatrial/atrioventricular block; DM, diastolic murmur; M, murmur; N, normal heart sound without murmurs or arrhythmias; SM, systolic murmur.

**Table 2:** Median, minimum, and maximum of total, Q1 and Q2 scores at T0 and T1 for each group (Group 1 = 15 students; Group 2 = 15 students).

Question’s Score	Group 1 T0	Group 1 T1	Group 2 T0	Group 2 T1
Total score (/18)	11.0 (5.0–14.0)	11.0 (8.0–16.0)	8.0 (4.0–12.0)	12.0 (6.0–15.0)
Q1 score (/10)	8.0 (5.0–10.0)	9.0 (6.0–10.0)	7.0 (3.0–10.0)	8.0 (5.0–10.0)
Q2 score (/8)	2.0 (0.0–6.0)	2.0 (1.0–6.0)	2.0 (0.0–6.0)	3.0 (1.0–5.0)

Abbreviations: Q1, first questions; Q2, second questions.

**Table 3:** Changes in total, Q1, and Q2 scores from T0 to T1 in Group 1 and Group 2 and results of the Group × Time interaction. Within-group changes are presented as mean change (T1–T0) with 95% bias-corrected and accelerated (BCa) bootstrap confidence intervals. Between-group differences represent the difference in mean change between Group 2 and Group 1, with 95% BCa bootstrap confidence intervals based on 2000 bootstrap samples. Partial eta squared (η^2^p) is reported as the effect size for the Group × Time interaction.

Outcome	Group 1 Mean Change (95% BCa CI)	Group 2 Mean Change (95% BCa CI)	Difference in Change G2–G1 (95% BCa CI)	Group × Time *F*(1,28)	*p*	η^2^p
Total score (/18)	0.67 (−0.47–1.80)	2.73 (0.96–4.50)	2.07 (−0.10–4.21)	3.73	0.064	0.118
Q1 score (/10)	0.73 (0.07–1.33)	1.47 (0.20–2.73)	0.73 (−0.72–2.20)	0.91	0.349	0.031
Q2 score (/8)	−0.07 (−1.00–0.80)	1.27 (0.47–2.00)	1.33 (0.22–2.47)	4.67	0.039	0.143

Abbreviations: Q1, first questions; Q2, second questions; BCa, bias-corrected and accelerated; CI, confidence intervals; η^2^p, partial eta squared.

## Data Availability

The original contributions presented in this study are included in the article. Further inquiries can be directed to the corresponding author.

## Supplementary Materials

The following supporting information can be downloaded at https://www.mdpi.com/article/10.3390/vetsci13090981/s1. Figure S1: Flow diagram of student enrollment, randomization, completion of the study protocol, and inclusion in the final analysis. Table S1: Participants’ responses to the feedback questionnaire administered at the end of the T1 assessment in Group 1 and 2.
