# Supplementary material for: Impact of Self-Directed Multimodal Digital Training on Veterinary Students’ Ability to Interpret Equine Heart Sounds
Source: Vet Sci. 2026 Sep 17;13(9):981. doi: 10.3390/vetsci13090981 (PMC13612010; doi:10.3390/vetsci13090981)
Supplement: Supplementary file 1 [file vetsci-13-00981-s001.zip › Supplementary Figure S1.pdf]

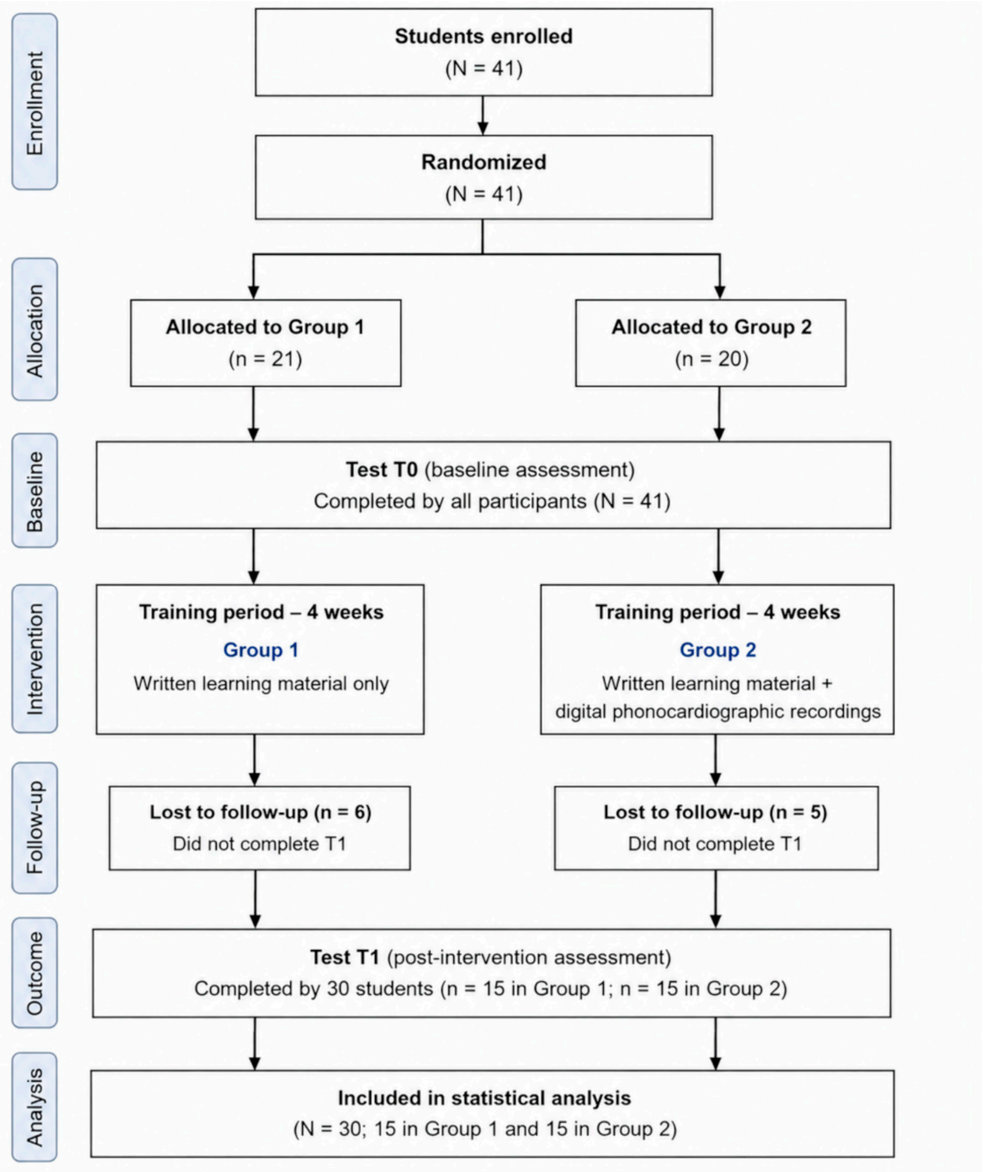

Figure S1. Flow diagram of student enrollment, randomization, completion of the study protocol, and inclusion in the final analysis.
