# Supplementary material for: Impact of Self-Directed Multimodal Digital Training on Veterinary Students’ Ability to Interpret Equine Heart Sounds
Source: Vet Sci. 2026 Sep 17;13(9):981. doi: 10.3390/vetsci13090981 (PMC13612010; doi:10.3390/vetsci13090981)
Supplement: Supplementary file 1 [file vetsci-13-00981-s001.zip › Supplementary Table S1.pdf]

Table S1. Participants' responses to the feedback questionnaire administered at the end of the T1 assessment in Group 1 and 2.

Questionnaire item	Group 1 (n = 15)	Group 2 (n = 15)
Time spent using the training material, n (%)		
< 1 hour	7 (46.7%)	5 (33.3%)
1 – 2 hours	7 (46.7%)	6 (40.0%)
2 – 4 hours	1 (6.7%)	4 (26.7%)
> 4 hours	0 (0%)	0 (0%)
Activity may improve teaching, n (%)		
Yes	15 (100%)	15 (100%)
No	0 (0%)	0 (0%)
Similar activities should be used in other subjects, n (%)		
Yes	15 (100%)	15 (100%)
No	0 (0%)	0 (0%)
Training satisfaction, n (%)		
0	0 (0%)	0 (0%)
1	4 (26.7%)	0 (0%)
2	3 (20.0%)	0 (0%)
3	2 (13.3%)	4 (26.7%)
4	4 (26.7%)	6 (40.0%)
5	2 (13.3%)	5 (33.3%)

Abbreviations: n, number.
